# Optimal Pandemic Influenza Vaccine Allocation Strategies for the Canadian Population

**DOI:** 10.1371/currents.RRN1144

**Published:** 2010-01-04

**Authors:** Ashleigh Tuite, David N. Fisman, Jeffrey C. Kwong, Amy Greer

**Affiliations:** ^*^Dalla Lana School of Public Health, University of Toronto; ^‡^Institute for Clinical Evaluative Sciences, Toronto, Ontario, Canada and ^§^Public Health Agency of Canada

## Abstract

Background: The world is currently confronting the first influenza pandemic of the 21st century. Influenza vaccination is an effective preventive measure, but the unique epidemiological features of swine-origin influenza A (H1N1) (pH1N1) introduce uncertainty as to the best strategy for prioritization of vaccine allocation. We sought to determine optimal prioritization of vaccine distribution among different age and risk groups within the Canadian population, to minimize influenza-attributable morbidity and mortality.

Methodology/Principal Findings: We developed a deterministic, age-structured compartmental model of influenza transmission, with key parameter values estimated from data collected during the initial phase of the epidemic in Ontario, Canada. We examined the effect of different vaccination strategies on attack rates, hospitalizations, intensive care unit admissions, and mortality. In all scenarios, prioritization of high-risk groups (individuals with underlying chronic conditions and pregnant women) markedly decreased the frequency of severe outcomes. Preferential vaccination of age groups at increased risk of severe outcomes following infection resulted in decreased mortality compared to targeting vaccine to age groups with higher transmission, at a cost of higher population-level attack rates. All simulations were sensitive to the timing of the epidemic peak in relation to vaccine availability, with vaccination having the greatest impact when it was implemented well in advance of the epidemic peak.

Conclusions/Significance: Our model simulations suggest that vaccine should be allocated to high-risk groups, regardless of age, followed by age groups at increased risk of severe outcomes. Vaccination may significantly reduce influenza-attributable morbidity and mortality, but the benefits are dependent on epidemic dynamics, time for program roll-out, and vaccine uptake.

## **Introduction**

The rapid global spread of a novel swine-origin influenza A (H1N1) (pH1N1) virus led the World Health Organization to declare an influenza pandemic on June 11, 2009  [1].  When there is a good match between circulating and vaccine strains, influenza immunization is the most effective preventive measure for reducing influenza-related morbidity and mortality [2].  Development of a vaccine against pH1N1 began in the early phases of the epidemic, leading to questions about prioritization of vaccine allocation within populations, given that not all vaccine will be distributed at once (due to production and logistical constraints). 

Seasonal influenza immunization campaigns typically target the elderly and those of any age with one or more underlying medical conditions, under the assumption that it is best to protect those most likely to have complications from influenza.  Recently, there has been debate over whether this is the best approach [3] [4].  The degree of protection conferred by the influenza vaccine appears to be lower in the elderly than in the general population [5] and it has been suggested that an immunization strategy based on reducing transmission would have a greater impact on reducing overall disease burden than the current practice of focusing vaccination efforts on at-risk groups [6] . In particular, the potential benefit of preferentially vaccinating school-aged children has been discussed, since this age group is disproportionately responsible for influenza transmission [7] [8] [9].  As with earlier pandemics, pH1N1 is characterized by age distributions that are distinct from those observed in seasonal influenza epidemics, with higher attack rates and increased proportionate mortality, in younger individuals [10] [11] [12].  This differential vulnerability to infection by age will have important implications for the choice of optimal vaccination strategies [13]. Given the uncertainty surrounding optimal vaccine allocation strategies and the unique epidemiological characteristics of pH1N1, we sought to determine optimal prioritization of vaccine distribution among different age groups in order to minimize influenza-attributable morbidity and mortality in the Canadian population.  To address this question we developed an age-structured mathematical model to describe expected pH1N1 transmission during the 2009-2010 influenza season.   We used this model to evaluate the optimal sequencing of vaccination allocation strategies.  Each strategy was tested using different assumptions relating to pre-existing immunity, vaccination coverage, and the timing of the epidemic peak. The outcomes of interest were influenza-attributable morbidity and mortality under different vaccination strategies.

## **Methods**

## Model structure

We developed a deterministic, age-structured compartmental model of influenza transmission in the Canadian population (see **Figure 1** for overall structure).  

**Figure:**
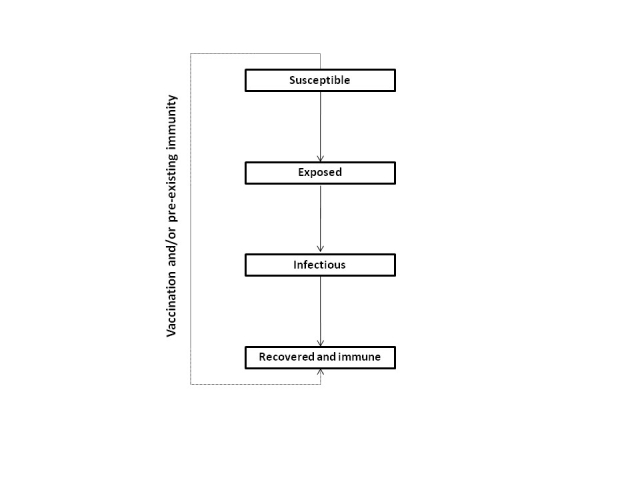
**Figure 1.  **Outline of model structure, showing population flows between compartments.  Each compartment is further stratified by age category (and by healthy and chronic condition states, where required).

The model ran from mid-April, 2009 (the date of the first identified cases of pH1N1 in Ontario, Canada) to June 30, 2010, representing a single influenza season.  As a result, we did not consider waning immunity following infection or vaccination, migration into or out of the population, or population aging.

The population was divided into four compartments representing different disease states: susceptible (*S*), exposed (*E*; i.e., infected but not infectious), infectious (*I*), and recovered (*R*).  Transmission of infection occurred through contact between susceptible and infectious individuals.  We assumed that 40% of infections were asymptomatic [14], but did not consider differential transmission in symptomatic versus asymptomatic cases.   

## Age structure and mixing patterns

To explore how vaccination of different age groups would impact overall influenza morbidity and mortality and to enable the representation of more realistic contact patterns within and between age groups, we included age stratification.  The population was divided into seven age classes with the following cutoffs:  0-4, 5-13, 14-17, 18-22, 23-52, 53-64 and ≥65.  Demographic information was obtained from 2006 Canadian census data [15].  We included the 53-64 year old age category to model the decreased susceptibility observed in persons born prior to the 1957 pandemic [12] [16] [17] and divided the younger ages according to school groupings to allow for the modeling of school-based vaccination programs.    Mixing within and between age strata was based on a population-based prospective study of contact patterns in eight European countries [18] .

For a subset of model scenarios, each age class was further subdivided into two states:  healthy or underlying chronic medical condition for which seasonal influenza immunization is recommended. Transitions between model compartments were identical for individuals in the healthy or chronic condition states, but probabilities of experiencing severe clinical outcomes were different.  We included a separate pregnancy state, representing women in the second or third trimester of pregnancy, with the number of women expected to be in this state at any given point in time derived using annual estimates of pregnancies and live births in Canada [19] [20].  

## Pre-existing immunity

To reflect the presence of immunity due to previous exposure to related influenza strains among individuals aged 53 and over, resistance to pH1N1 was modeled by moving some individuals from the susceptible to the resistant compartment at time zero.  Since it is currently clinically impractical to distinguish individuals with pre-existing exposure to the circulating strains, we assumed that they received the same vaccination coverage as the susceptible population (i.e. there was no way to preferentially immunize the truly susceptible population).  

## Vaccination 

Vaccination with two doses of H1N1 vaccine was modeled by removing a select number of individuals from the susceptible compartment immediately following administration of the second dose of vaccine.  Vaccination began in mid-November (November 15^th^), with a delay of 21 days between administration of the first and second doses.  The fraction of the vaccinated population that acquired immunity was based on vaccine effectiveness estimates of 70 percent; for a given age group, with a vaccine effectiveness (*VE*) and coverage (*C*), the proportion removed from the susceptible to the resistant compartment was VE*C.  We assumed that this group was fully protected against infection, with the remaining fraction VE*(1-*C*) receiving no protection.  Although this does not reflect the true situation, where most vaccinated individuals will experience some degree of protection, this approach has been used previously and has been demonstrated to provide a reasonable model of partial efficacy [21].  We did not consider the effect of partial protection following the first dose.  We assumed that it took four weeks to administer the first dose of vaccine to all age groups and vaccine allocation within each targeted sub-group (described below) occurred simultaneously at the beginning of each week.

## Disease natural history and model parameterization

Model parameters for pH1N1 were based on the currently available case data from the province of Ontario (**Table 1**) [22].  A range of estimates of the proportion of the population aged ≥53 with pre-existing immunity  to pH1N1 influenza was derived from reported serological data [23], the relative risk of infection by age observed in Ontario [12] and model calibration to the Ontario epidemic curve. 

**Table 1.**  Model Parameter Values.

**Figure:**
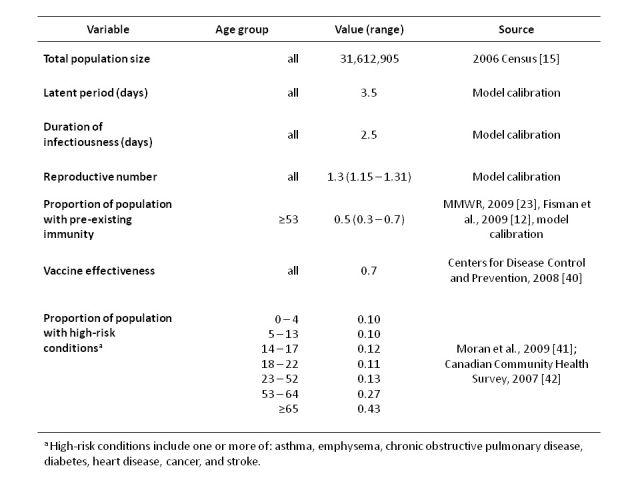

Age-specific hospitalization, ICU admission, and mortality rates were calculated using data from Ontario’s Integrated Public Health Information System (iPHIS), which collected information on all laboratory-confirmed cases of pH1N1 in the province reported between April 13 and June 21, 2009 (**Table 2**) [22].  To account for expected under-ascertainment of less severe cases, we multiplied the denominator (total cases) by a factor of ten when calculating hospitalization and case-fatality rates [24].

**Table 2**.  Estimated Rates of Hospitalization, ICU Admission, and Mortality by Age and Risk Group for pH1N1 in Ontario, April to June 2009. 

**Figure:**
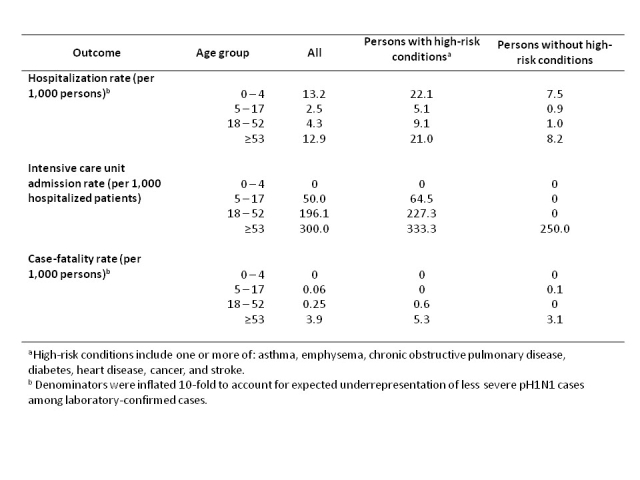

We used vaccination coverage data for the province of Ontario [25] [26], which operates a universal influenza immunization program that provides influenza vaccine free of charge to the entire population aged six months or older, as a base case for H1N1 vaccine uptake (**Table 3**).  Telephone survey data on willingness to accept H1N1 vaccine in the province of Ontario obtained using the province’s Rapid Risk Factor Surveillance System (RRFSS) [27] was used as an upper bound of vaccine uptake in the Canadian population (Ruth Sanderson, Ontario Agency for Health Protection and Promotion, personal communication).  Age-specific data on underlying chronic conditions were obtained from the 2007 cycle of the Canadian Community Health Survey [25]. 

**Table 3**.  Influenza Vaccination Coverage Levels. 

**Figure:**
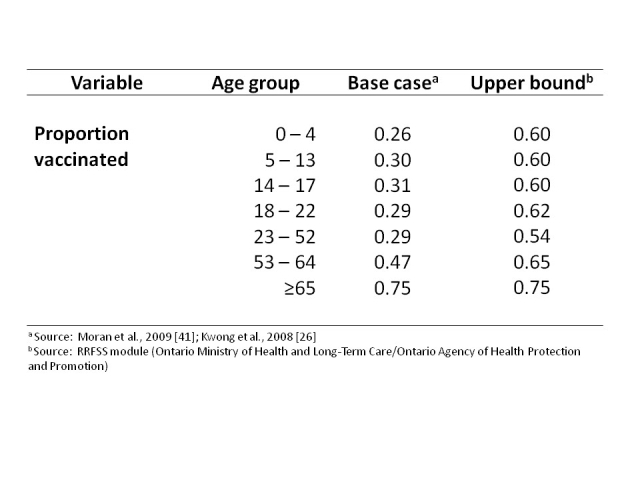

To model the impact of assumptions about the dynamics of H1N1 transmission over the course of the summer, where typical influenza seasonality and changes in contact patterns may reduce the basic reproductive number (R0), we modified R0 to generate differential timing of the peak of the epidemic curve.  We considered the effect of different vaccination strategies when the epidemic peak occurred in October (no change in R0 over the summer), November (R0 decreases but remains above endemic levels from July to September), December (R0 = 1 from July to September), or January (R0 = 1 from July to October).  We also adjusted R0 to account for different levels of pre-existing immunity to pH1N1 in the population (i.e., to give the same* effective* reproductive number under different immunity assumptions).

## Vaccination scenarios

We considered four vaccination strategies.  For all scenarios, the total number of vaccine doses was not a limiting factor; adequate supply of vaccine was available for all individuals requiring immunization [28].

 

1.  Attack rate-based strategy (AR): Vaccine distributed first to age groups with the highest model-predicted attack rates (order of vaccine allocation by age group: 5-17, 18-52, 0-4, ≥53)
2. Outcome-based strategy (Outcome): Vaccine distributed first to age groups at the highest risk of a severe outcome, defined as hospitalization, ICU admission, or death, following infection with pH1N1 (order of vaccine allocation by age group: ≥53, 18-52, 0-4, 5-17).
3. and 4. Risk-based strategy (High risk/AR or High risk/Outcome) :  Vaccine preferentially distributed to individuals of any age with an underlying risk condition (based on seasonal influenza recommendations [2]) and pregnant women (in the second or third trimester), followed by an attack rate- or outcome-based strategy described above (delayed by one week to allow for immunization of high-risk groups first).

## Model calibration

The model was calibrated to fit the initial epidemic curve observed in Ontario.  Data for laboratory-confirmed cases with a reported exposure date between April 13 and June 1, 2009 were obtained from iPHIS.  Travel history data, including illness on return to Mexico, were used to model the observed multiple introductions of pH1N1 into the Ontario population early on in the pandemic.  

## Sensitivity analyses

We tested the robustness of model projections to baseline assumptions and parameter values by performing sensitivity analyses, with model inputs varied over plausible ranges, and incorporating alternate assumptions regarding vaccine program attributes.  We evaluated the effect on model outputs of changing the time period for delivery of the first dose of vaccine to the entire population to two or six weeks, switching to a single vaccine dose, reducing vaccine effectiveness in the ≥65 age group (across a range of effectiveness of 30-60 percent), and varying the proportion of asymptomatic cases.   

## **Results**

### Initial epidemic dynamics and model calibration

The model appeared well-calibrated to epidemic curves for pH1N1 influenza and matched the initial transmission dynamics observed in Ontario (**Figure 2**).

**Figure:**
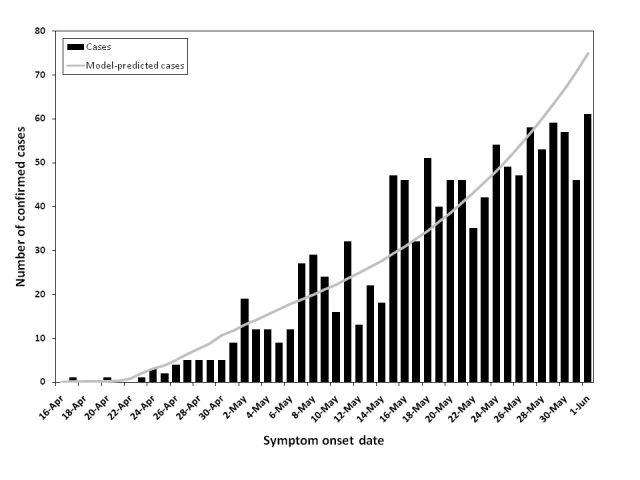
**Figure 2.  **Confirmed cases of locally-acquired pH1N1 in Ontario by symptom onset date, April 16 – June 1, 2009.  Cases that reported a history of travel to Mexico prior to illness onset are not included.  Model-predicted cases assuming 50 percent pre-existing immunity in the ≥53 age group, R0 of 1.3, latent period of 3.5 days, and duration of infectiousness of 2.5 days are shown (line).

Figure 3 illustrates the pH1N1 infection dynamics generated by the model; epidemic curves peaked in different months, depending on assumptions made about influenza transmission behaviour during the summer months, but overall attack rates were consistent across model runs for a given estimate of pre-existing immunity in the pre-1957 cohort.  In the absence of vaccination, the average infection attack rate across the entire Canadian population was 35.1% (range 33.2 – 36.8%).  Age-specific patterns of influenza transmission reflected typical mixing patterns within a population, with epidemic curves peaking first in younger age groups, followed by the elderly.

**Figure:**
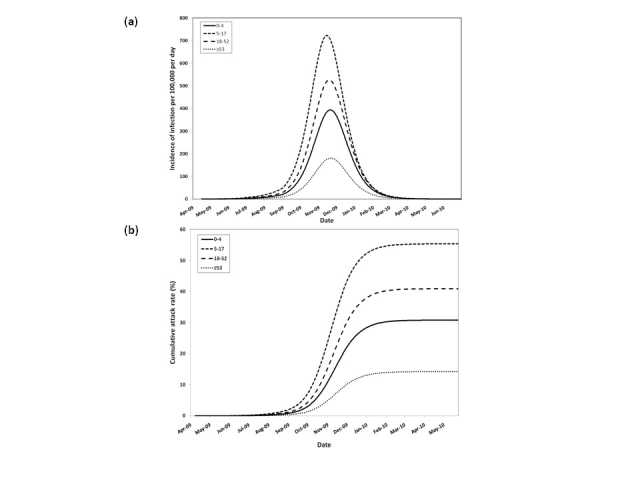
**Figure 3.**  (a) Simulated age-stratified daily pH1N1 infection incidence per 100,000 population and (b) age-specific attack rates between April 2009 and June 2010, in the absence of vaccination or other interventions.  Both symptomatic and asymptomatic cases are shown.  The curves are based on an assumption of fifty percent pre-existing immunity in the ≥53 age group and a decrease in R0 from 1.3 to 1.15 between July and September. 

### Effect of timing of epidemic peak in relation to vaccine availability on outcomes

            Given the uncertainty around pH1N1 dynamics over the coming months and timelines for vaccine delivery, we investigated the impact of the timing of the epidemic peak on whether an attack rate- or outcome-based vaccination strategy was preferred (**Figure 4**).  For an October peak, neither approach is likely to significantly alter outcomes.  For each month that the epidemic is delayed, there is enhanced effectiveness of all vaccination strategies.

**Figure:**
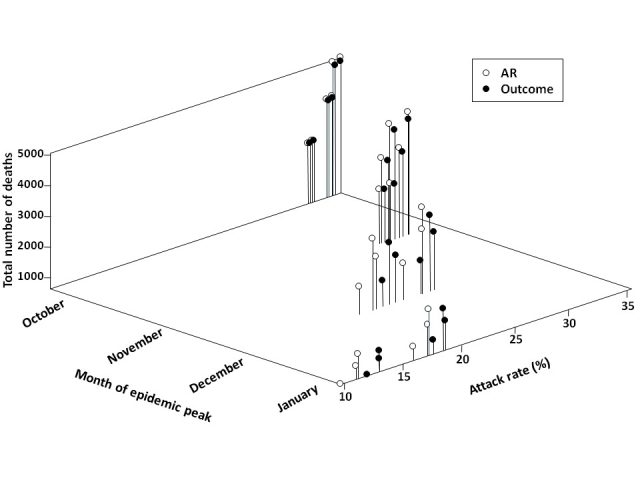
**Figure 4.  **Effect of timing of epidemic peak on preferred vaccination strategy.  Total model-predicted attack rates and deaths by month of the pandemic peak are shown, when implementing attack rate (AR)-  or outcome-based vaccination strategies.  For each month of the epidemic peak, outcomes are presented for three values of pre-existing immunity among individuals aged ≥53 (30%, 50%, and 70%) and two vaccination coverage levels (base case and upper bound).  For all scenarios, vaccination campaigns are initiated on November 15, 2009.

### Attack rate-based versus outcome-based vaccination strategies

We evaluated the percent reduction in predicted attack rates, number of hospitalizations, ICU admissions, and deaths under these two strategies, relative to the no intervention scenario.  The attack rate-based strategy was most effective at reducing the total number of infections and minimizing hospitalizations when the epidemic peaked in December or January, with minimal difference in the impact of competing strategies on overall attack rates when the epidemic peaked earlier.  When there was 30 percent pre-existing immunity in the individuals born prior to 1957 group, there was no preferred strategy for minimizing hospitalizations.  Using ICU admissions as the outcome of interest, the outcome-based strategy was preferred when there were low levels of pre-existing immunity, but there was no advantage to using one strategy over the other when immunity in the older age groups was >50 percent.  By contrast, when mortality was assessed as the endpoint of interest, an outcome-based strategy was preferred to an attack rate-based strategy for any combination of values for pre-existing immunity and vaccine coverage, with the exception of the assumption of 70 percent immunity to pH1N1 in individuals aged >53 combined with a January peak.  Under this latter scenario, there was no difference between strategies.

### Prioritization of vaccine delivery to individuals with underlying high-risk conditions (risk-based strategy)

We assessed the effect of preferentially immunizing individuals of any age with an underlying medical condition, prior to implementing an attack rate- or outcome-based strategy.  Despite the resulting delay in vaccine allocation to the remaining population, for all scenarios, hospitalizations and ICU admissions were reduced compared to vaccination strategies that did not target high-risk groups (**Figure 5**).  When the epidemic peak occurred in December or January, this approach had a less marked effect in reducing mortality and resulted in higher cumulative attack rates than strategies that did not prioritize high-risk groups, whereas for an October or November epidemic peak, this approach had a larger effect in reducing mortality.

**Figure:**
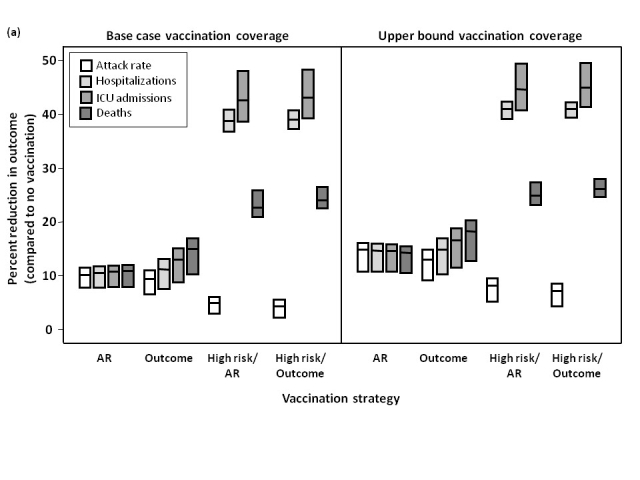

**Figure:**
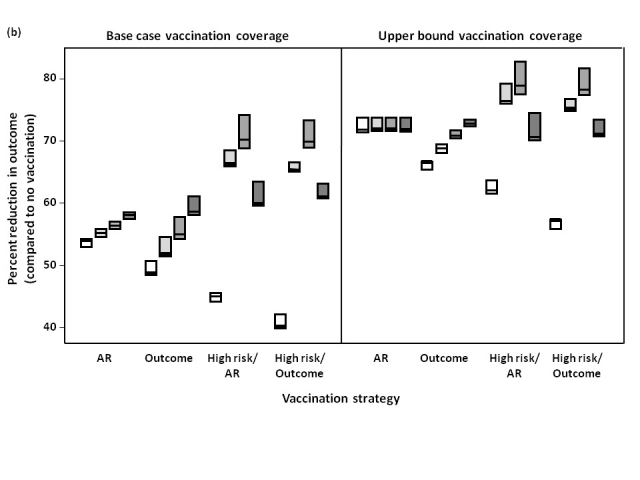
**Figure 5.  **Percent reduction in attack rate, hospitalizations, ICU admissions, and total deaths, relative to no vaccination, under different vaccination strategies.  The effectiveness of different strategies was evaluated assuming an epidemic peak in (a) November, 2009 or (b) January, 2010, with vaccination campaigns initiated on November 15, 2009.  Results for October, 2009 and December, 2009 were similar to November, 2009 and January, 2010, respectively, and are not shown.  The impact of vaccination coverage is also shown, with base case rates representing the lower bound of vaccine uptake in the Canadian population, compared to likely upper limits of vaccine uptake.  The midpoint of the boxes represents the median percent reduction in the outcome of interest, with the upper and lower bounds representing the maximum and minimum reductions, respectively, under varying assumptions of pre-existing immunity in individuals aged ≥53 (i.e., 30%, 50%, or 70%).  Details of the different vaccination strategies (AR, Outcome, High risk/AR, High risk/Outcome) are outlined in the Methods. 

**

### Sensitivity analyses

Increasing the length of time to administer the first vaccine dose in all age groups from two to six weeks decreased the effectiveness of vaccination programs when the epidemic peak was in December or January, but did not have an effect when the peak occurred earlier (**Figure 6**). 

**Figure:**
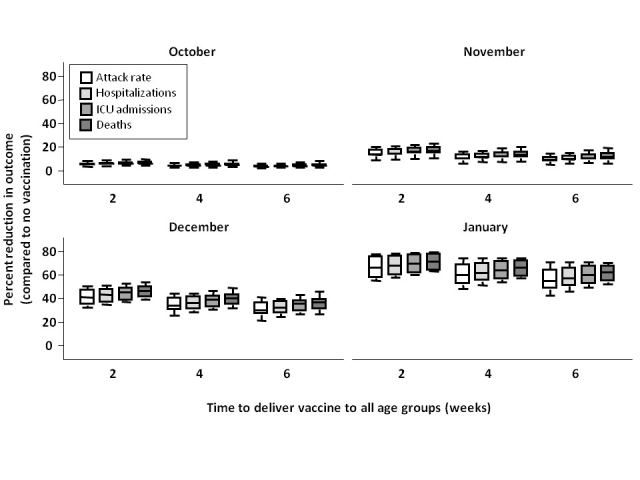
**Figure 6.**  Percent reduction in attack rate, hospitalizations, ICU admission, and total deaths, relative to no vaccination with different program roll-out lengths.  Outcomes were assessed assuming that time to administration of a single dose of vaccine to all age groups was  2, 4, or 6 weeks, with vaccination campaigns commencing on November 15, 2009.  Estimates are pooled across vaccination strategy used (attack rate- and outcome-based), vaccination coverage (base case and upper bound), and levels of pre-existing immunity in individuals aged ≥53 (30%, 50%, and 70%) and are shown by month of epidemic peak. 

Reducing vaccine effectiveness in individuals aged ≥65 did not have a marked effect on the ranking of vaccination strategies when levels of pre-existing immunity in the pre-1957 group were 30 or 50 percent.  With 70 percent pre-existing immunity and lower bound vaccination coverage, reducing vaccine effectiveness to 60 percent or lower resulted in the attack rate-based strategy becoming favoured over outcome-based, when evaluating total deaths as the outcome of interest.  Lowering vaccine effectiveness did not alter the ranking of the strategies when other outcomes (ICU admissions, hospitalizations, or attack rate) were the endpoints of interest.

Emerging data suggest that a single dose of vaccine may be sufficient to confer protective immunity against infection with pH1N1 [29] [30].  When we tested the impact of a single dose on outcomes, we found no qualitative differences in the rank-order of vaccination strategies under different conditions for the majority of scenarios.  However, for a January epidemic peak with high vaccination coverage, the attack rate-based strategy was more attractive than the outcome-based strategy, regardless of which endpoint was evaluated. 

**Increasing the proportion of infectious individuals with asymptomatic influenza reduced the absolute number of cases experiencing severe outcomes, but did not change the relative rankings of the different strategies. 

## **Discussion**

We used a mathematical model to evaluate optimal pH1N1 vaccination strategies, focusing our analysis on the Canadian population and considering the effect of targeting different age groups for prioritization of vaccine allocation on projected hospitalizations and mortality.  Depending on the outcome assessed and the assumptions used, both attack rate- and outcome-based strategies were effective in reducing morbidity and mortality, but in most scenarios, delaying vaccine distribution by one week to preferentially immunize individuals with underlying high-risk conditions was the optimal strategy.  We observed that the dynamics of pH1N1 transmission is a critical area of uncertainty, with all vaccination strategies having limited impact if the epidemic peak occurs prior to or concomitantly with vaccine availability (projected for mid-November).   

Our analysis focused on the occurrence of severe outcomes and did not directly consider the effect of vaccination on reducing disease transmission and the resultant downstream effects, such as reduced societal disruption and economic costs (such as those associated with time lost from work or school).  Additionally, when assessing severe outcomes, there is a need to consider how these outcomes may interact; for instance, a strategy that focuses on reducing mortality at the expense of higher attack rates could lead to the saturation of ICU capacity, resulting in higher mortality in younger age groups than has been observed to date.

The epidemiology of pH1N1 appears distinct from that of seasonal influenza (but similar to that of prior pandemics [11] [31] ) in that younger age is associated with the highest attack rates, a phenomenon that has resulted in a higher absolute burden of morbidity and mortality in this age group than is typically observed with seasonal influenza, even though per-case risks of poor outcome may not differ from those seen with seasonal influenza.  However, although older age groups are less likely to be infected with the pandemic strain than younger individuals, infections in individuals aged > 50 years documented in Ontario have been associated with increased ICU admissions and death [22].

Our model assumes that two doses of pH1N1 vaccine will be required to elicit a protective response, but emerging data have demonstrated that a single dose may be sufficiently immunogenic in healthy adults [29] [30] .  Whether these results extend to children, the elderly, or individuals with underlying medical conditions remains to be seen.  The implications of a single dose vaccine are similar to shifting the epidemic peak to later in the winter, resulting in enhanced effectiveness for any vaccination strategy adopted, relative to a two-dose schedule.  The preference for an attack rate-based strategy using a single vaccine dose when vaccination coverage is high agrees with a recent study suggesting that targeting age groups at the highest risk of infection may be the optimal solution [32], but in our model, this is only the case when  vaccine is available well before the epidemic peak.  Finally, we evaluated the impact of poor vaccine effectiveness in older individuals on preferred strategies, as this has been a concern with seasonal vaccine [33]; we found limited impact of decreased effectiveness on the rank-ordering of preferred strategies except when older individuals were highly likely (70%) to be immune to infection in the absence of vaccination, and were effectively “pre-vacccinated” by early life influenza exposures. 

Our analysis is subject to several important limitations.  As with all mathematic models, this model includes simplifyingassumptions and incorporates parameter values that are subjectto some uncertainty.  Model calibration to existing data was used to derive estimates of key epidemiologic parameters and these values are in agreement with estimates from other settings [34] [35].  We incorporated non-homogeneous mixing patterns between age groups, but did not consider the effect of spatial heterogeneity.  However, other studies have demonstrated that estimates of R0 appear to be consistent across locations and spatial scales [36] [37].  Some other simplifying assumptions included non-differential transmissibility of influenza by symptomatic and asymptomatic cases and non-incorporation of other concurrent mitigation strategies on influenza transmission, including antivirals and social distancing measures, on influenza transmission.  We also did not consider the impact of co-circulating seasonal influenza strains, although recent data suggest that reduced circulation of seasonal strains may be observed in the upcoming influenza season [38] [39].  To address the uncertainty in our estimates of mortality and hospitalization rates, due to both the low frequency of occurrence of these outcomes and reporting biases and other limitations inherent in surveillance data, we have focused our analysis on qualitative results.   

In summary, we have developed an age-structured mathematical model to evaluate optimal vaccination strategies for pH1N1.  This model demonstrates the importance of the interaction between pH1N1 transmission dynamics and the demographic characteristics of population at risk of pH1N1 infection on the potential effectiveness of vaccination strategies.  It also highlights the value of moving away from strictly age-based vaccination prioritization schemes toward strategies that target high-risk groups, regardless of age.   

### **Acknowledgements**

We thank Michael Campitelli  and Ruth Sanderson for providing data, and Barbara Law and Susan Tamblyn for helpful discussions related to plausible vaccination strategies.

The opinions, results and conclusions reported in this paper are those of the authors and are independent from the funding sources. No endorsement by the Institute for Clinical Evaluative Sciences or the Ontario Ministry of Health and Long-Term Care is intended or should be inferred.

## Funding information

AT receives support from the Mathematics of Information Technology and Complex Systems (MITACS) Accelerate Program through both MITACS funding and a matching contribution from the Ontario Agency for Health Protection and Promotion. DF is supported by an Early Researcher Award from the Ontario Ministry of Research and Innovation and is supported by the Canadian Consortium for Pandemic Preparedness Modelling, MITACS and the CIHR.  JK was supported by an Ontario Ministry of Health and Long-Term Care (MOHLTC) Career Scientist Award, a University of Toronto Department of Family and Community Medicine Research Scholar Award, and the Institute for Clinical Evaluative Sciences (ICES). 

The opinions, results and conclusions reported in this paper are those of the authors and are independent from the funding sources. No endorsement by the Institute for Clinical Evaluative Sciences, Public Health Agency of Canada or the Ontario Ministry of Health and Long-Term Care is intended or should be inferred.  

## Competing interests

DF received matching funds from Sanofi-Pasteur for an Ontario Early Researcher Award on pertussis epidemiology.  Sanofi-Pasteur manufactures a pandemic influenza vaccine not distributed in Canada. No competing interests declared by the other authors. 
